# Visual complexity of egg patterns predicts egg rejection according to Weber's law

**DOI:** 10.1098/rspb.2022.0710

**Published:** 2022-07-13

**Authors:** Tanmay Dixit, Andrei L. Apostol, Kuan-Chi Chen, Anthony J. C. Fulford, Christopher P. Town, Claire N. Spottiswoode

**Affiliations:** ^1^ Department of Zoology, University of Cambridge, Cambridge, UK; ^2^ Computer Laboratory, University of Cambridge, Cambridge, UK; ^3^ DST-NRF Centre of Excellence at the FitzPatrick Institute of African Ornithology, University of Cape Town, Rondebosch, Cape Town, South Africa

**Keywords:** coevolution, visual complexity, avian brood parasitism, Weber's law, receiver perception, mimicry

## Abstract

Visual complexity is ubiquitous in nature. Drivers of complexity include selection in coevolutionary arms races between antagonists. However, the causes and consequences of biological complexity and its perception are largely understudied, partly because complexity is difficult to quantify. Here, we address this by studying egg pattern complexity and its perception in hosts (tawny-flanked prinia *Prinia subflava*), which visually recognize and reject mimetic eggs of their virulent brood parasite (cuckoo finch *Anomalospiza imberbis*). Using field data and an optimization algorithm, we compute a complexity metric which predicts rejection of experimentally placed conspecific eggs in prinia nests. Real cuckoo finch eggs exhibit significantly lower pattern complexity than prinia eggs, suggesting that high complexity benefits hosts because it distinguishes host eggs from parasitic eggs. We show that prinias perceive complexity differences according to Weber's law of proportional processing (i.e. relative, rather than absolute, differences between stimuli are processed in discrimination, such that two eggs with simple patterns are more easily discriminable than two with complex patterns). This may influence coevolutionary trajectories of hosts and parasites. The new methods presented for quantifying complexity and its perception can help us to understand selection pressures driving the evolution of complexity and its consequences for species interactions.

## 1. Introduction

Biology is replete with examples of visual complexity [1], which can be loosely defined as how difficult a pattern is to reproduce [2]. Patterns which appear complex and difficult to reproduce (to the human eye) can be present on the bodies of organisms, such as common cuttlefish (*Sepia officinalis*), or on external structures constructed by organisms, such as mate-attracting bowers made by bowerbirds. Theoretical models have suggested that coevolution can drive the evolution of complexity, in particular when arms races drive reciprocal evolution in antagonists [3–5]. For instance, in host–parasite arms races, selection from parasitic mimics (forgeries) can drive the evolution of complex ‘signatures of identity’ in hosts [6–8], just as counterfeiters have driven banks to create more complex banknotes which are difficult to forge. In a sexual selection context, complex visual signals may be favoured due to mate choice or competition, as a result of antagonistic coevolution, or because they convey honest information about mate quality or species identity [9]. While these and other adaptive hypotheses have been proposed as biological conditions under which visual complexity should be elevated [10], they have not been empirically tested, and nor have hypotheses about how organisms might perceive complexity. Indeed, though complexity has received considerable attention in fields such as engineering, mathematics and computer science, it is in biology—where arguably the most complex systems are found—that complexity and its perception have been neglected [11].

This neglect is at least in part because complexity is difficult to measure objectively and in a biologically relevant way [12]. Although the mathematician Andrey Kolmogorov proposed an objective, information theoretical measure of complexity, defined as the minimum size of programme required to recreate the object, Kolmogorov complexity is not computable [2,13]. Therefore, other measurements of complexity are required. Quantifying complexity is challenging because many different traits may contribute to complexity [2,12], and because sensory systems may vary in how they process complexity. A simple method to measure complexity is to ask large numbers of people to rank images by visual complexity, with consistently high rankings indicating high complexity [14]. However, this method introduces human bias [15] when the intended receivers of complex stimuli are non-human animals. More recently, however, other methods of quantifying complexity have been developed, which have aimed to take into account the sensory system and psychology of receivers [1,2,13,16]. For measures of visual complexity to be biologically relevant, they must be based on receiver perception. Therefore, an ideal model system for experimental tests of hypotheses to explain the evolution of biological complexity would be one in which receiver perception of complexity, and the traits which contribute to complexity, can be objectively quantified.

**Figure 1.**
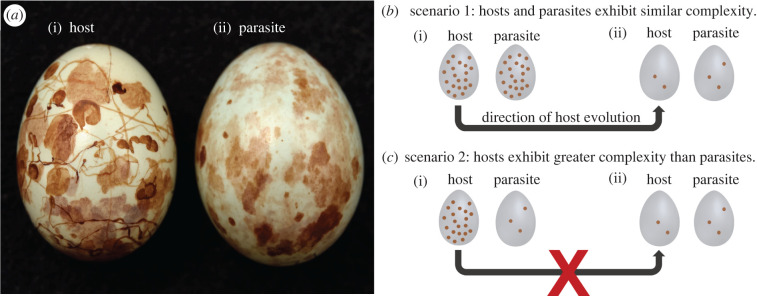
(*a*) (i) A prinia egg and (ii) a cuckoo finch egg, illustrating the complex patterning present in both species. (*b*,*c*) A schematic illustrating expected evolutionary trajectories of hosts depending on whether parasites exhibit similar complexity to hosts. As the real eggs in (*a*) illustrate, high complexity is not just produced by more markings on eggs, but other traits such as the variation in, and unpredictable distribution of, those markings. However, for simplicity, we depict the magnitude of pattern complexity as the number of spots on eggs. More spots indicate higher complexity (i.e. higher stimulus magnitude). (*b*) Scenario 1: parasitic egg patterns are good matches in complexity to host egg patterns, as depicted in (i). Even though both pairs of eggs (i) and (ii) differ in only one spot, the difference between the two is easier to recognize in (ii). This illustrates Weber's law; discrimination is easier in (ii) because the *relative* difference in the number of spots is larger than in (i). Therefore, all else being equal, hosts will evolve reduced complexity, since this increases discriminability of eggs. Parasites would evolve to better mimic this reduced complexity (rightmost egg). (*c*) Scenario 2: host eggs exhibit more complex patterns than parasitic eggs, as depicted in (i). Hosts would not benefit from evolving reduced complexity (ii) because they would be becoming more similar to parasites, and therefore less discriminable regardless of whether their perception adheres to Weber's law. Therefore, Weber's law should only lead hosts to evolve towards lower stimulus magnitudes if hosts do not exhibit higher stimulus magnitudes than parasites. (Online version in colour.)

Egg mimicry by avian brood parasites offers a study system meeting these requirements. Avian interspecific brood parasites lay eggs in the nests of host species, often imposing considerable costs on hosts [17]. Hosts have therefore evolved defences, including egg rejection which has in turn selected for egg mimicry by parasites [17]. Host egg phenotype diversification reduces the probability of accurate mimicry by parasites, leading to individual ‘signatures of identity’ in host eggs [6–8]. Some hosts exhibit highly complex egg signatures [18–20] (figure 1*a*). Because host eggs are the models for their parasitic mimics, parasitic eggs may in turn evolve complex patterning [18,19] (figure 1*a*). Given that host ability to discriminate between measurable complex phenotypes can be quantified using egg rejection experiments, brood parasitism provides an excellent system in which to study selection pressures acting on visual complexity.

There is a general consensus that egg pattern (signature) complexity should be beneficial for hosts, because complex signatures are expected to be more difficult to forge [19,21,22]. For example, immaculate host eggs lack all pattern complexity, and therefore a parasitic egg must simply match the host's background colour to be an effective mimic [23]. Yet, high complexity may also carry costs to hosts. For example, among hosts of the common cuckoo *Cuculus canorus*, signatures of intermediate complexity exhibited optimal (i.e. high) recognizability and therefore discriminability, since more complex signatures carried a cost of reduced signature recognizability [19].

In this study, we devise a biologically relevant metric of complexity in a brood parasitic system and subsequently consider a second potential cost to hosts of high complexity, which could arise if hosts proportionally process differences in complexity. Weber's law of proportional processing states that the relative difference (as opposed to the absolute difference) between stimuli predicts discriminability [24,25]. This means that if receiver discrimination adheres to Weber's law, the ‘just noticeable difference’ (i.e. the smallest difference between two stimuli that can be discriminated) is proportional to stimulus magnitude. Therefore, if complexity is a stimulus used in discrimination, then two simple phenotypes (i.e. low magnitude of complexity) will be more easily discriminated than two complex phenotypes (i.e. high magnitude of complexity) [26].

Although Weber's law has been applied to a range of taxa and biological contexts (reviewed in [27]), it has rarely been tested in coevolutionary systems. In deceptive coevolutionary mimicry systems such as the aggressive egg mimicry of brood parasites, models (i.e. hosts) benefit from increased discriminability in their stimuli. Therefore, under Weber's law, models (here, host eggs) should benefit from lower stimulus magnitudes since lower magnitude stimuli are easier to discriminate [26]. If complexity is a stimulus that is used by hosts in decision-making, therefore, Weber's law could provide a second mechanism selecting against the evolution of highly complex signatures in hosts (figure 1*a*). However, reduced host signature complexity should be selected for only when parasitic eggs are good matches in complexity to host eggs (or, irrespective of Weber's law, when parasitic eggs have more complex patterns than host eggs). By contrast, when parasitic egg patterns are less complex than host egg patterns, hosts should not benefit from reduced complexity, since this would make host eggs more similar to parasitic eggs (figure 1*b*). Thus, the influence of Weber's law on the evolutionary trajectories of hosts should depend on whether host eggs are more or less complex than parasitic eggs.

Here, we conducted the first experimental study of complexity in a brood parasite–host system, and tested whether hosts process complexity according to Weber's law. We studied an Afrotropical system: the tawny-flanked prinia *Prinia subflava* (hereafter ‘prinia’) has evolved a diverse array of egg colours and seemingly complex egg signatures that facilitate rejection of mimetic eggs of its parasite, the cuckoo finch *Anomalospiza imberbis* [28]. We carried out field experiments on host rejection of foreign eggs and used these to compute a biologically relevant complexity score based on higher-level attributes of pattern features quantified by a computer vision tool for analysing visual patterns, NaturePatternMatch [19]. We then tested whether Weber's law applies to how hosts process complexity, using the framework proposed in [26]. Finally, we tested for differences in pattern complexity between prinia and cuckoo finch eggs, to assess whether visual complexity provides useful information to hosts in detecting a parasitic egg, and to determine whether high complexity should be costly for hosts.

## 2. Methods

### (a) Field experiments

Field experiments (*n* = 126) were carried out on Semahwa Farm (around 16°74′ S, 26°90′ S) and surrounding areas in the Choma District of southern Zambia during January to April 2018–2020. The experimental protocol was almost identical to that in [28]. Because cuckoo finches typically remove one or more host eggs when they lay their own, we replaced (rather than adding) one egg from a prinia nest with a conspecific egg (the ‘experimental egg’). Experiments were carried out during the first half of the incubation period, after the full clutch had been laid (incubation stage was assessed by holding a torch under the egg to visualize the embryo, following [29]). Hosts lay clutches of 2–4 eggs (modal clutch size = 3). All eggs in the host clutch, along with the experimental egg, were measured with digital calipers and photographed (see below). Nests were monitored daily for 4 days or until the host rejected the experimental egg. If the egg was not rejected within 4 days, it was recorded as accepted. Four days is a conservative threshold in this system since almost all rejected eggs are removed within 3 days [28].

Unlike in [28], where hosts were given both subjectively good and subjectively poor matches to their eggs, experimental eggs were chosen to always be a good match (to the human eye) of the host clutch phenotype. We did this to focus on effects of complex pattern differences rather than egg colour or simpler pattern measures. We therefore expected a lower rate of rejection in the present experiments, than the approximately 50% rejection rate estimated in [28].

### (b) Photography of clutches

For each experiment, the entire host clutch and the experimental egg were photographed in linearized RAW format. Photographs were taken in the field in shade using a Nikon D90 camera with a 60 mm Micro-Nikkor lens. ISO (400) and aperture (*f*/8) were kept constant and shutter speed varied to control exposure. Two grey standard squares (N6.5 and N5; reflectance values 36.2% and 19.8%, respectively) of an X-Rite ColorChecker Passport (X-Rite, MI, USA) were used to normalize images. To capture the full pattern of the egg, each egg was rotated three times by 90 degrees around the long axis, resulting in four images (‘sides’ *a*, *b*, *c* and *d*, where *a* is opposite *c*, and *b* is opposite *d*). For all pattern metrics calculated, the values for all four sides of an egg were highly repeatable, with high intra-class correlation coefficients (all greater than 0.81; electronic supplementary material, table S1).

### (c) Image analysis

Using the MICA toolbox [30] in ImageJ [31], images were normalized and scaled to 29 px/mm; eggs were individually ‘cut out’ (i.e. removed from the background) and masked (i.e. given an artificial black background). Greyscale images were produced using the green channel, chosen because this channel corresponds closely to the sensitivity of avian double cones, thought to be involved in pattern processing [32]. Pattern features were extracted using NaturePatternMatch (NPM) [19] (see [18] for details). Briefly, the scale-invariant feature transform (SIFT) algorithm used in NPM detects and encodes local features (SIFT features) as 132-dimensional vectors, including the position, scale and principal orientation of features. Alongside other traits (§2d), variation in these dimensions can contribute to pattern complexity, since high variation in these dimensions would make a pattern less predictable.

### (d) Complexity trait measurement and coefficient optimization

Pattern complexity can be described by a combination of the number of elements in a pattern, and the variation and unpredictability among those elements [12]. Thus, we measured six traits describing pattern complexity: (i) number of pattern features; (ii) variation in the position of features; (iii) variation in the scale (size) of features; (iv) variation in the orientation of features; (v) Redies change, a measure of how much intensity (i.e. brightness) changes across an image; and (vi) group metric, a measure of clustering tendency of features and within-cluster feature variation. All of these except for the Redies change were based on features extracted using NPM (electronic supplementary material, §1). Complexity was defined as a weighted sum of the six traits. Using an optimization algorithm (electronic supplementary material, figure S1), we optimized the coefficients of the six traits such that the difference in complexity between experimental (conspecific) egg patterns and host egg patterns best predicted egg rejection. Full details of the calculation and optimization of the complexity metric are provided in the electronic supplementary material (§1); briefly, the number of features, the variation in their position, and the Redies change contributed most to the optimized complexity metric. We did not include any other colour or pattern metrics in the optimization, to focus solely on complexity. Complexity had a linear axis of magnitude (electronic supplementary material, figure S2).

As an independent validation of whether differences in our complexity metric predicted egg rejection in a different dataset, and to confirm that our metric was not overfitted to the 2018–2020 data, we also tested whether differences in complexity predicted rejection in a previously published dataset from 2007 to 2009 [28]. This dataset was from the same species and study site, and used nearly identical field experimental methods (electronic supplementary material, §4). Images from 2007 to 2009 were initially scaled to 50 px mm^−1^ [28] and then scaled down to 29 px mm^−1^ for comparability in this study. Furthermore, given that hosts were given a range of colour matches in 2007–2009, only experiments in which the experimental egg was a good match in colour to the host clutch were included in this validation (electronic supplementary material, §4). The 2007–2009 and 2018–2020 datasets could only be analysed separately, due to differences in image processing (e.g. different normalization methods; electronic supplementary material, §4). Therefore, we used the 2007–2009 data only for validation, rather than combining the two datasets.

### (e) Statistical analysis

When calculating the average complexity of host clutches, removed eggs were excluded from the averages, following [28]. Average measurements for full clutches were highly correlated with averages excluding the removed egg (electronic supplementary material, table S1).

Rejection was modelled using logistic regression (function glm) in R v. 4.0.2 [33]. First, the complexity score for each egg was calculated as the average complexity score of side *a* and side *c*, since these two sides do not overlap and therefore an average score of the two estimates the complexity of the entire pattern. The absolute difference between the complexity of the experimental egg and the mean complexity of the host clutch (hereafter, ACD) was used as a predictor to confirm that difference in complexity predicted rejection.

Then, relative complexity difference (hereafter, RCD) was calculated as (ACD/mean complexity of host clutch). RCD was highly correlated with ACD (Pearson's *r* = 0.862, CI_0.95_ = 0.808 to 0.902). Following [34], we nevertheless conducted logistic regression using RCD and ACD, particularly as the variance inflation factors in the model containing ACD and RCD as predictor variables were relatively low (3.16 for each variable).

If RCD is a better predictor of discrimination than ACD, then this implies that the stimulus is proportionally processed. We therefore included both ACD and RCD in the full model to test which was the better predictor. Some sensory systems adhere to the ‘near-miss’ to Weber's law, where receiver discrimination is affected less by stimulus magnitude than predicted by Weber's law [35–38]; others are thought to adhere to the ‘opposite-miss’ to Weber's law, where receiver discrimination is affected more by stimulus magnitude than predicted by Weber's law [37,39,40]. These other forms of processing can be distinguished from Weber's law by testing whether discrimination is not only predicted better relative differences between stimuli than absolute differences between stimuli, but also predicted by absolute *magnitudes* of the stimuli [40]. Specifically, if absolute magnitudes predict discrimination along with relative differences, then the near- or opposite-miss is supported (depending on whether the coefficient of absolute magnitude is positive or negative). If absolute magnitudes do not predict discrimination but relative differences do, then Weber's law is supported. Here, the absolute magnitude of the stimulus is the mean complexity of the host clutch ('host complexity'; hereafter HC). Therefore, the full model included ACD, RCD and HC as fixed effects (following [40]).

Our models did not include the colour and ‘lower-level’ pattern metrics previously shown to predict rejection in this system [18,28], because in our study, experimental eggs were chosen by eye to be good matches to host clutches in these traits. We subsequently validated that this was so, and confirmed that colour and lower-level pattern metrics did not predict rejection in the present dataset (electronic supplementary material, §5).

Percentage of variation explained by each model was calculated using Nagelkerke's *R*^2^ (package rsq in *R*). If models contained multiple predictor variables, percentage of variation explained by each predictor was calculated using hierarchical partitioning (package hier.part in R). We carried out three tests to determine whether host perception of egg complexity conformed to Weber's law, or to the near- or opposite-miss to Weber's law:

First, model comparison was carried out using the R package *MuMIn* [41], and models compared using Akaike information criteria (AICc) [42] and Bayesian information criteria (BIC) [43].

Second, likelihood ratio tests were carried out to compare models in which the predictor variables were correlated.

Third, we note that both ACD and RCD can be written as

$$\frac{\left|a-b\right|}{b^{k}}\;\mathrm{expression}\;1$$

where *a* is the complexity of the experimental egg and *b* is the average complexity of the host clutch. When *k* = 0, expression 1 equals |*a − b*|, which is ACD; when *k* = 1, expression 1 equals |a−b|/b, which is RCD. Furthermore, the near-miss and opposite-miss to Weber's law can also be described by expression 1: the near-miss is described by 0 < *k* < 1 [35,37] and the opposite-miss by *k* > 1. Thus, by estimating the value of k, we can evaluate how complexity is processed. We used this re-parametrization to compare predictive models with different values of *k* from −1 to 2 at intervals of 0.25. If hosts process complexity according to Weber's law (i.e. they process RCD), then the minimum of a curve plotting AIC against k should be at around *k* = 1. If hosts process absolute differences (i.e. ACD), then the minimum should be at around *k* = 0. To find the minimum, a fifth-order polynomial was fitted to the points, and the value of *k* corresponding to minimum AIC was estimated using the uniroot.all function in *R*.

For all the above models, the sample size was *n* = 119 (*n* = 19 rejected; *n* = 100 accepted). Although 126 experiments were conducted, there were six females on which two experiments were carried out, since females re-nest during seasons. One of each pair of duplicates was randomly selected and excluded from the analysis. We also excluded one experiment where the host clutch size was 1, since complexity excluding the replaced host egg cannot be calculated for a clutch size of 1. In all analyses, predictor variables were scaled (by subtracting the mean and dividing by the standard deviation); all reported estimates and standard errors pertain to scaled variables. In figures, all predictors shown are unscaled.

## 3. Results

### (a) Absolute complexity difference predicts egg rejection

**Figure 2.**
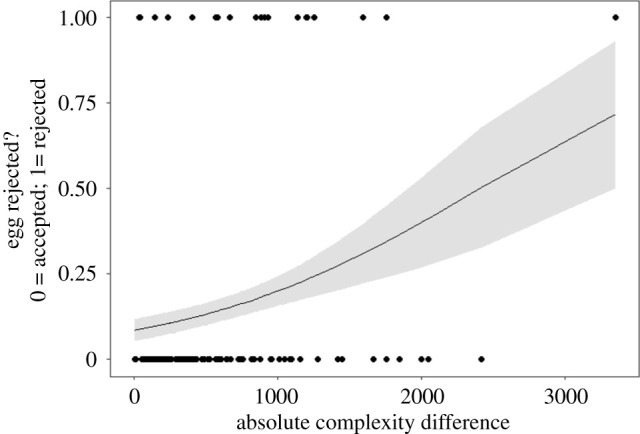
ACD between experimental ‘parasitic’ egg and host clutch predicts egg rejection. Points show individual field experiments; the curve plots predicted rejection probabilities according to the model Rejection ∼ ACD; shading indicates standard errors.

We first verified that the complexity measure computed and optimized using egg rejection data did indeed predict egg rejection. ACD significantly predicted rejection (estimate ± s.e. = 0.544 ± 0.222, d.f. = 117, *Z* = 2.448, *p* = 0.014, Nagelkerke's *R*^2^ = 0.09; figure 2), meaning that experimental eggs which differed more from the average complexity of the host clutch were more likely to be rejected. Our measure also predicted rejection in the independent 2007–2009 dataset which was not used to train the optimality model (estimate ±s.e. = 1.109 ± 0.492, *Z* = 2.254, *p* = 0.024, *R*^2^ = 0.12; electronic supplementary material, §4), suggesting that the measure was not overfitted to the 2018–2020 dataset.

### (b) Relative complexity difference is a better predictor of egg rejection than absolute complexity difference

We then tested whether RCD was a better predictor of rejection than ACD, using three lines of evidence.

**Figure 3.**
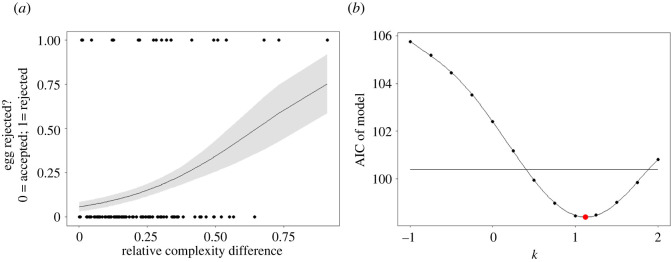
Egg rejection based on pattern complexity conforms to Weber's law. (*a*) RCD between the experimental egg and host clutch predicts egg rejection in field experiments. Points show individual experiments; the curve plots predicted rejection probabilities according to the model Rejection ∼ RCD; shading indicates standard errors. (*b*) AICs for models of the form Rejection ∼ $|a-b|/b^{k}$, for −1 < *k* < 2, where a is the complexity of the parasitic egg and b is the complexity of the host clutch. The curve is a fitted polynomial of order five, showing that the minimum AIC (large red point) is estimated to be at *k* = 1.127. The intersections between this curve and the black line (indicating *y* = 2 + (minimum AIC)) give the 95% confidence intervals of 0.402 and 1.88 around this ‘best’ value of *k*. (Online version in colour.)

First, from a full model containing ACD, RCD and HC (electronic supplementary material, table S2), the best model according to both AICc and BIC was a model with RCD as the only predictor (for this model, estimate ± s.e. = 0.730 ± 0.238, d.f. = 117, *Z* = 3.068, *p* = 0.002, *R*^2^ = 0.14; figure 3*a*). This means that host perception of complexity is best described by Weber's law. Furthermore, perception does not adhere to the near-miss or opposite-miss, because the final model did not include HC as a significant predictor of rejection.

Second, we used likelihood ratio tests to compare the models Rejection ∼ ACD and Rejection ∼ RCD to the model Rejection ∼ ACD + RCD. The addition of RCD to a model with ACD as the only predictor improved the model ($\chi_{1}^{2}=4.06,$ *p* = 0.044), whereas the addition of ACD to a model containing RCD as the only predictor did not improve the model ($\chi_{1}^{2}=0.094,$ *p* = 0.759); this again suggests that RCD is a better predictor than ACD.

Third, we plotted the AIC of models where we varied the value of *k* in expression 1 (see 'Methods'). The minimum AIC value was at *k* = 1.127 (AIC = 98.389). Thus, the lowest AIC model was close to *k* = 1, again suggesting that RCD is the best predictor (figure 3*b*). Ninety-five per cent confidence intervals (calculated by adding two to the minimum AIC) were at *k* = 0.402 and *k* = 1.88; these exclude *k* = 0, further demonstrating that discrimination adheres to Weber's law.

### (c) Prinia egg patterns are more complex than cuckoo finch egg patterns

**Figure 4.**
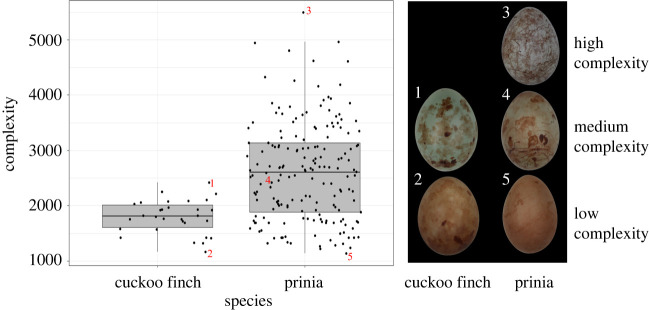
Prinia egg patterns are significantly more complex than cuckoo finch egg patterns. Images of cuckoo finch (1, 2) and prinia (3–5) eggs, corresponding to numbered points on the boxplots, illustrate examples of eggs with high (3), medium (1,4) and low (2,5) complexity. No cuckoo finch eggs of high complexity were observed. Note that eggs vary in colour; in our experiments, colour variation between host and experimental eggs was deliberately minimized. (Online version in colour.)

Prinia egg patterns were significantly more complex than cuckoo finch egg patterns (Kruskal–Wallis *χ*^2^ = 24.22, d.f. = 1, *p* < 0.001; figure 4). There is a consistent difference between host and parasite eggs in the complexity metric even though this metric was derived from experimental data involving only hosts.

## 4. Discussion

In this study on complexity and its perception, we derived a metric of pattern complexity which predicts egg rejection by a common host of the brood-parasitic cuckoo finch. Using this metric, we found evidence that Weber's law of receiver perception operates in host rejection decisions in this system, since the relative difference in complexity between host and an experimental ‘parasitic’ egg (the egg of another host female) was a better predictor of rejection than the absolute difference in complexity. This is the first demonstration that Weber's law can apply to rejection of eggs by the host of a brood parasite. Finally, we showed that host egg patterns are more complex than those of their parasite.

### (a) Absolute complexity difference predicts rejection

The absolute difference in complexity between host and experimental egg patterns predicted egg rejection, as expected given that the metric was optimized to predict rejection. However, the metric also predicted rejection in an independent dataset from the same species and study site (from 2007 to 2009), with a different distribution of host–parasite differences, and for which it was not optimized. Furthermore, even an optimized complexity metric could have been a poor predictor of rejection if the traits contributing to complexity were not used in rejection, and therefore this study demonstrates that measures of complexity can be shown to have biological relevance. Variation in ACD predicted 9–12% of variation in egg rejection, leaving much variation unexplained. In this system, it has been shown that pronounced differences in colour and lower-level features (such as marking size) predict approximately 32% of the variation in egg rejection [18,28]. This suggests that when simpler differences between eggs exist (i.e. not in the current study which was designed to exclude them), they may be preferentially used by hosts as signals of egg identity; indeed, the low rejection rate observed in this study is probably because experimental eggs were chosen to be good matches to the host clutch. Furthermore, the median prinia egg complexity is approximately 720 units greater than the median cuckoo finch egg complexity (figure 4), and the probability of egg rejection at a difference of 720 units is low (approximately 16%; figure 2), suggesting that most parasite eggs would be accepted on the basis of complexity. Again, this illustrates that other colour and pattern differences are likely to be more important in explaining rejection in this system, though complexity differences have a small but significant effect.

We do not suggest that prinias directly compute the complexity of egg patterns; rather, complexity is likely to be a proxy for integrative aspects of egg phenotype processed by the host when comparing eggs. This is the case for any calculated pattern metric, since we do not fully understand how visual systems process pattern [1]. The finding that complexity significantly predicts rejection, even when individual lower-level features are unable to do so, emphasizes the importance of ‘higher-level differences' in addition to colour and lower-level features in signalling egg identity [18].

The finding that hosts use complexity in discrimination makes biological sense because hosts exhibited significantly higher complexity than parasites (figure 4) and because complexity shows high repeatability within clutches, with an ICC value of 0.76 (electronic supplementary material, table S1), which is higher than ICCs of other pattern traits in prinias [44]. Both of these properties mean that complexity provides reliable information about egg identity. Previous studies in several systems (e.g. [28,45]) have similarly found that traits that consistently differ in traits between hosts and parasites are those that best predict rejection, presumably because they provide reliable information.

Prinias may have evolved high complexity because this reduces the mimetic fidelity of relatively simple cuckoo finch eggs. Indeed, parasitized species of warblers (Cisticolidae, including the tawny-flanked prinia) and weavers (Ploceidae) show greater ‘entropy’ (randomness or unpredictability) in egg phenotypes than unparasitized species [20]. Entropy has been used as a measure of complexity of other stimuli, such as auditory signals in birds and dolphins [46,47]. For hosts of brood parasites, an increase in complexity (measured as entropy) is thought to allow diversification into areas of phenotypic space unoccupied by parasites and to produce signatures that are difficult to forge [20,48]. However, entropy is a population-level measure, whereas our complexity metric is an individual-level measure, so these different measures of complexity are not interchangeable although they seek to capture a similar biological principle. Importantly, an individual-level measure is required to test hypotheses about benefits and costs of complexity, as we do here.

Why might cuckoo finch egg patterns be less complex than prinia egg patterns? This may simply be due to evolutionary lag, for instance if cuckoo finches are constrained from evolving complex phenotypes by their genetic architecture. The cuckoo finch parasitizes multiple host species [44], such that lineages parasitizing specific host species (gentes) must maintain the ability to mimic a specific host despite interbreeding between males and females of different gentes. This problem is solved in the cuckoo finch by maternal inheritance via the female-specific W chromosome, preventing the costs of recombination and segregation when parasites from different gentes interbreed [49]. However, maternal inheritance carries the cost of slower evolution, constraining the evolution of complexity by forgoing recombination and heterozygosity [49,50]. By contrast, prinias have autosomal inheritance of egg phenotype, such that they retain its advantages for generating novelty and diversity in coevolutionary arms races [49]. Maternal inheritance may therefore explain the relatively simple egg pattern phenotypes exhibited by cuckoo finches. A non-mutually exclusive explanation for simpler egg patterns in cuckoo finches is that their closest relatives (*Vidua* spp.) lay immaculate white eggs [51]. This suggests that cuckoo finches have more recently evolved egg patterns than prinias, whose closest relatives (*Prinia* and *Cisticola* spp.) lay patterned eggs [52]. Perhaps, therefore, there has been insufficient evolutionary time for cuckoo finches to evolve high complexity, though as previously shown in this system, frequencies of traits can change rapidly [53].

Our method of quantifying complexity could be applied to other systems in which organisms differentiate between stimuli. A complexity metric optimized for one system will probably be suboptimal for another. Therefore, psychophysical experiments are needed to derive a relevant measure of complexity (optimized from chosen traits) which predicts discrimination between stimuli in each specific system. Researchers can choose any trait that *a priori* seems to measure complexity; for instance, the number of pattern components, the variation in properties of these components, and/or how unpredictable the components are [12]. It is necessary to be transparent about which traits are chosen prior to optimization and be aware that important traits may not have been considered.

### (b) Relative complexity difference is a better predictor of rejection than absolute complexity difference

An understanding not only of what stimuli organisms perceive, but *how* they perceive them, is needed to understand the selection pressures acting on senses and signals [54]. In sensory ecology, we usually assume that organisms perceive absolute differences between stimuli [27]. However, Weber's law of proportional processing states that relative rather than absolute differences between stimuli predict discrimination between them [24,25]. Here, we showed that host discrimination of complexity adheres to Weber's law, and therefore that the relative difference is a more important trait to measure than the absolute difference. Furthermore, host discrimination follows Weber's law rather than the near- or opposite-miss to Weber's law [37,40,55]. Although we studied complexity because complex patterning remains understudied, Weber's law could also apply to other traits processed by hosts, for which it must be tested independently. For Weber's law to apply, traits must have certain properties such as having quantitative magnitudes on continuous or quasi-continuous scales [26]. This rules out traits such as colour differences between host and parasitic eggs, since although differences in colour between eggs can be calculated, an egg does not have a quantifiable (i.e. numerical) magnitude of colour.

Weber's law is known to be evolutionarily relevant in foraging and mate choice [27,56,57], but this is one of the first demonstrations of Weber's law applying to receiver discrimination in a *coevolutionary* system. In coevolutionary systems, receiver perception may influence the evolution of both models and mimics, and can inform expectations about evolutionary trajectories of multiple interacting species [26]. Weber's law states that discriminability declines as stimulus magnitudes increase, because for a given absolute stimulus difference, the relative stimulus difference is greater at a low-stimulus magnitude. Since hosts benefit from having egg signatures that are discriminable from parasitic forgeries, under certain circumstances (i.e. when parasitic eggs are good matches to host eggs in complexity, or more complex than host eggs), hosts should benefit from less complex signatures [26]. This runs counter to the general assumption that hosts should benefit from ever more complex signatures [19,21,22].

Should hosts therefore evolve towards lower complexity in this system? We show that this should not occur, because cuckoo finch egg patterns are less complex than prinia egg patterns. Therefore, prinias should not *currently* be expected to evolve towards lower complexity, since this would make host eggs more similar to parasitic eggs (figure 1*b*). Only if cuckoo finches ‘catch up’ to prinias in complexity, should prinias benefit from and thus evolve reduced complexity (figure 1*a*). Instead, in this system, parasitic eggs should experience selection for more complex patterns, since this would improve mimetic fidelity. Host eggs should either remain at an elevated level of pattern complexity, or evolve greater complexity that would further reduce mimetic fidelity. The prediction that hosts should evolve towards lower magnitude stimuli should therefore be tested in systems where the trait that is proportionally processed has high mimetic fidelity.

Although Weber's law operates in egg discrimination, as revealed by our field experiments using conspecific eggs with high complexity, Weber's law may have little impact on whether hosts reject real parasitic eggs. Since parasitic eggs exhibit lower pattern complexity than host eggs, in many cases, differences in complexity could be perceived regardless of whether hosts perceive absolute or RCD between egg patterns [26]. Our experiments therefore provide evidence for Weber's law that natural observations of rejection of parasitic eggs would probably have missed.

## 5. Conclusion

We computed a biologically relevant measure of pattern complexity and found evidence that host perception of differences in complexity adheres to Weber's law. Our method produced a receiver-specific metric of complexity, which is important because complexity is difficult to define objectively, and because different receiver species will probably process complexity differently. This method can be applied to other animal signals to test whether specific traits contribute to biological complexity, facilitating the study of the evolution of both complex signals and the sensory systems that receive them. In our study system, host egg patterns are significantly more complex than those of parasitic eggs, and therefore hosts are not currently expected to evolve towards lower complexity as Weber's law would otherwise lead us to expect. Instead, hosts may experience selection for greater complexity, or relaxed selection because their eggs are already highly discriminable from parasites. Furthermore, parasites should experience selection for greater complexity over time. We suggest that the prediction that hosts should evolve towards reduced stimulus magnitudes should be tested in parasite–host systems where mimetic fidelity is higher. Since the latter prediction only applies when host perception adheres to Weber's law (or the near- or opposite-miss to Weber's law), host perception itself must also be studied across systems. While much work on receiver perception focuses on sensory systems, greater understanding of perceptual processes such as Weber's law will improve our understanding of how mimicry and pattern complexity might evolve. More generally, measuring complexity and how organisms perceive it can improve our understanding of the selection pressures driving the evolution of biological complexity, and its consequences on species and their interactions.

## Data Availability

Data and R code associated with this manuscript are available on Dryad Digital Repository at https://doi.org/10.5061/dryad.nvx0k6dvh [58]. Code for quantifying complexity is available at https://github.com/andreiapostol/eggcomplexity. Further details of methods and results are provided in the electronic supplementary material [59].
